# Identification of a universal antigen epitope of influenza A virus using peptide microarray

**DOI:** 10.1186/s12917-020-02725-5

**Published:** 2021-01-07

**Authors:** Qiuxia Wang, Zhihao Sun, Jingzhi Li, Tao Qin, Hongwei Ma, Sujuan Chen, Daxin Peng, Xiufan Liu

**Affiliations:** 1 College of Veterinary Medicine, Yangzhou University, 48 East Wenhui Road, Yangzhou, Jiangsu 225009 People’s Republic of China; 2 Jiangsu Co-Innovation Center for the Prevention and Control of Important Animal Infectious Disease and Zoonoses, Yangzhou, 225009 Jiangsu People’s Republic of China; 3 Jiangsu Research Centre of Engineering and Technology for Prevention and Control of Poultry Disease, Yangzhou, 225009 Jiangsu People’s Republic of China; 4 Division of Nanobiomedicine, Suzhou Institute of Nano-Tech and Nano-Bionics, Chinese Academy of Sciences, Suzhou, 215000 People’s Republic of China; 5 Joint Laboratory Safety of International Cooperation of Agriculture & Agricultural-Products, Yangzhou, Jiangsu 225009 People’s Republic of China

**Keywords:** Influenza virus, Epitope, Broad-spectrum, Peptide, Microarray

## Abstract

**Background:**

Hemagglutinin is a major surface protein in influenza A virus (IAV), and HA2 is relative conserved among different IAVs. It will be meaningful to identify broad-spectrum epitopes based on the HA2 protein.

**Results:**

Overlapping peptides of the HA2 protein of the H5N1 IAV A/Mallard/Huadong/S/2005 were synthesized and loaded on modified silica gel film to form a microarray, and antisera against different subtypes of IAVs were used to screen universal epitopes. The selected epitope was further confirmed by western blotting using anti-peptide immune serum and viruses rescued with amino acid substitution. The results showed that 485-FYHKCDNECME-495 of the H5 14th peptide in HA2 had broad-spectrum binding activity with antisera against H1, H3, H4, H5, H6, H7, H8, H9, and H10 subtype IAV. Substitution of amino acids (K or D) in rescued viruses resulted in decreased serum binding, indicating that they were critical residues for serum binding activity. In Immune Epitope Database, some epitopes containing 14–4 peptide were confirmed as MHC-II-restricted CD4 T cell epitope and had effects on releasing IL-2 or IFN.

**Conclusion:**

The identified epitope should be a novel universal target for detection and vaccine design and its ability to generate immune protection needs further exploration.

**Supplementary Information:**

The online version contains supplementary material available at 10.1186/s12917-020-02725-5.

## Background

Influenza A virus (IAV), a highly infectious zoonotic and variable pathogen, presents a substantial threat to public health worldwide, causing huge economic losses in the poultry industry owing to its high morbidity and mortality [1, 2]. Since 1918 influenza H1N1 virus killed more than 50 million people, influenza virus has been receiving more and more attention [3]. Influenza viruses could infect not only animals but also humans, and there was the possibility of interspecies transmission. Study shows that the highly pathogenic H5N1 avian influenza virus causes millions of deaths in poultry and cross-species infection in human [4]. These factors have posed a huge pressure to the surveillance of influenza viruses and prevention of influenza.

IAV is negative-strand RNA virus and belongs to the family *Orthomyxoviridae*. It is composed of eight gene segments that encode at least 17 different kinds of protein [5–7]. Hemagglutinin (HA) is main envelope glycoprotein and can be cleaved into the HA1 and HA2 subunit. Continual mutations result in vast changes in HA proteins and as a consequence, cross-immunity between different subtypes is extremely poor. Thus far, 18 HA subtypes have been identified, which can be phylogenetically segmented into two large groups—Group 1 (H1, H2, H5, H6, H8, H9, H11, H12, H13, H16, H17, and H18) and Group 2 (H3, H4, H7, H10, H14, and H15) [8, 9]. Hence, effective universal epitopes are of great significance for the control of influenza virus.

Currently, vaccination is still considered the most effective and powerful means against IAVs, and the HA1 subunit plays a major role in immune response induced by traditional vaccines. However, immune pressure brings out mutations of HA1. As a result, it is difficult for conventional vaccines to deal with the new strains with mutations, due to lack of cross-immune protection [10]. On the contrary, HA2 subunit is located in stem and highly conserved in different subtypes of IAV. Antibodies against conformational or linear epitopes found in the HA2 are more broadly neutralizing and protective [11].

Several methods have been developed to identify conserved epitopes. Lohia et al identified three peptides containing T cell epitopes from the Matrix 1 protein of the H1N1 influenza virus using the immunoinformatics-based consensus approach, which were conserved (> 90%) among different subtypes of IAV and might to be promising candidates for universal vaccine design [12]. Ichihashi et al predicted six cytotoxic T lymphocyte (CTL) epitopes from internal proteins of the H5N1 highly pathogenic avian influenza by peptide prediction programs; three of which were protective and highly conserved among three different IAV subtypes [13]. Reverse-deriving epitopes by preparing numbers of monoclonal antibodies has been sought after by many people [14–16]. Li et al used four monoclonal antibodies, which can neutralize the HA of H7N9, H3N2, and H9N2 to recognize novel linear epitopes by peptide-based ELISA [17]. Zhu et al identified six critical amino acid positions (92, 145, 166, 167, 168, and 197) in H9 antigenic sites based on the reactivity of variant and wild-type strains with monoclonal antibodies [18]. Some conserved epitopes also have been identified in HA2 of H5 subtype IAV [19]. In this study, novel conserved epitopes in HA2 of IAV were screened by peptide microarray and antisera against different subtypes of IAVs.

## Results

### Identification of peptides with broad-spectrum serum binding activity

**Fig. 1:**
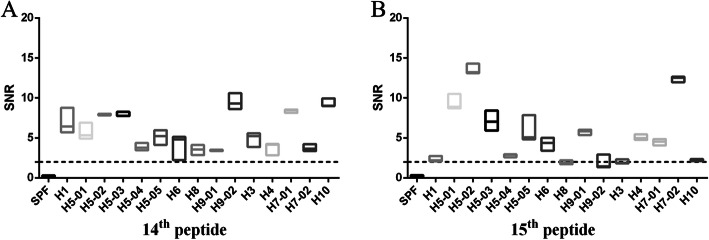
The SNRs of the reaction between sera against different subtypes of IAVs and H5 14th peptide (**a**) or H5 15th peptide (**b**). Synthetic peptides were sampled onto iPDMS (modified silica gel film) to form chip, sera were diluted 1:50 (v/v) with serum dilution buffer for binding activity assay. Signal-to-noise ratio (SNR, SNR = (signal strength–background intensity)/background intensity) was determined by using GenePix Pro 6.0 software. The dotted line represents the SNR = 2. H5–01: serum against A/Mallard/Huadong/S/2005; H5–02: serum against A/Duck/Huadong/wx1205/2016; H5–03: serum against A/Goose/Huadong/yz1111/2016; H5–04: serum against 2.3.4.4d vaccine strain; H5–05: serum against 2.3.2.1d vaccine strain; H7–01: serum against A/Chicken/Jiangsu/W1–8/15; H7–02: serum against A/Chicken/Huadong/JD/17; H9–01: serum against A/Chicken/Shanghai/F/98; H9–02: serum against A/Chicken/Taixing/10/2010. Each reaction was repeated for 3 times, SNR values were expressed as means ± standard deviation. X axis, sera against different subtypes of IAVs; Y axis, SNR values

To discover serum binding activity of peptides, the chip containing 18 overlapping peptides from the HA2 protein of the H5N1 subtype IAV S strain was reacted with 15 antisera against 9 subtype IAVs. The results showed that the SNR (Signal-to-noise ratio, SNR = ((signal strength-background intensity)/background intensity) of reactions between the 1st, 5th, 6th, 7th, 8th, 18th, or 19th peptides and antisera were all lower than 2, indicating that there was no binding activity between these peptides and antisera (Additional file 1 (Fig. S1-S4), Additional file 2). The 2nd, 3rd, 4th, 12th, 13th, 16th, or 17th peptides showed binding activity to partial sera against the H5 subtype IAVs (Additional file 1 (Fig. S1-S4), Additional file 2). In contrast, H5 14th and 15th peptides not only showed positive binding activity to different serum samples against self-subtype IAVs, but also showed positive binding activity to sera against H1, H3, H4, H6, H7, H8, H9 or H10 subtype IAVs, indicating that the two peptides had broad-spectrum binding activity (Fig. 1, Additional file 2). As general binding activity of the H5 14th peptide to different sera was higher than that of the H5 15th peptide, and there was partial overlapping between two peptides, the H5 14th peptide (KELGNGCFEFYHKCDNECME) was selected for further study.

**Fig. 2:**
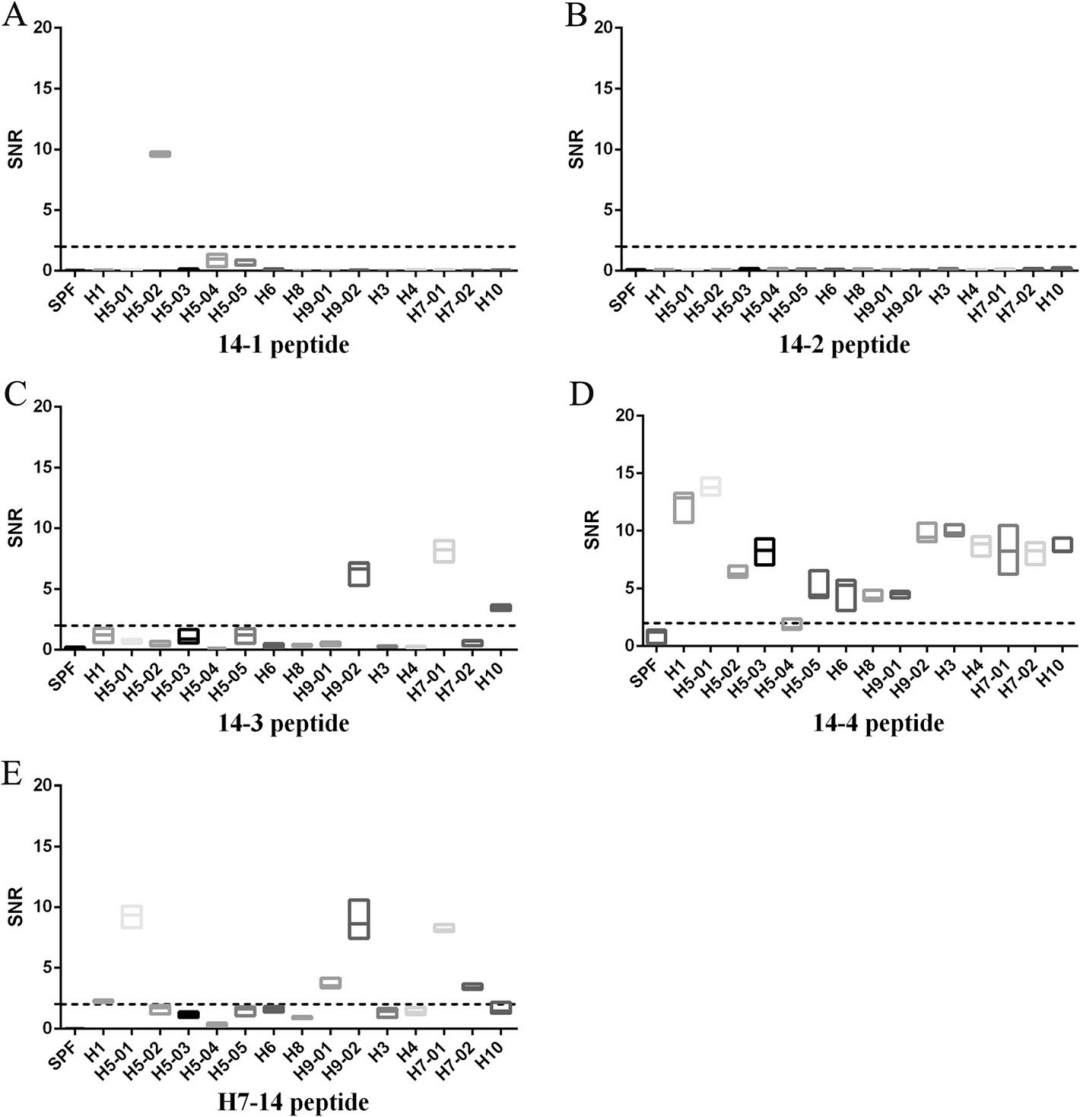
The SNRs of the reaction between sera against different subtypes of IAVs and H5 14–1 (**a**), H5 14–2 (**b**), H5 14–3 (**c**), H5 14–4 (**d**) or H7–14 (**e**) peptides. Synthetic peptides were sampled onto iPDMS (modified silica gel film) to form chip, sera were diluted 1:50 (v/v) with serum dilution buffer for binding activity assay. Signal-to-noise ratio (SNR, SNR = (signal strength–background intensity)/background intensity) was determined by using GenePix Pro 6.0 software. The dotted line represents the SNR = 2. H5–01: serum against A/Mallard/Huadong/S/2005; H5–02: serum against A/Duck/Huadong/wx1205/2016; H5–03: serum against A/Goose/Huadong/yz1111/2016; H5–04: serum against 2.3.4.4d vaccine strain; H5–05: serum against 2.3.2.1d vaccine strain; H7–01: serum against A/Chicken/Jiangsu/W1–8/15; H7–02: serum against A/Chicken/Huadong/JD/17; H9–01: serum against A/Chicken/Shanghai/F/98; H9–02: serum against A/Chicken/Taixing/10/2010. Each reaction was repeated for 3 times, SNR values were expressed as means ± standard deviation. X axis, sera against different subtypes of IAVs; Y axis, SNR values

To verify whether the 14th peptide at the same position of other subtype IAVs had broad-spectrum serum binding activity, a peptide from the HA2 protein of the H7 subtype IAV was synthesized and subjected to the microarray assay. The results showed that the H7–14 peptide bound well with sera against H7 subtype IAVs, and several kinds of H5 and H9 subtype IAVs (Fig. 2E, Additional file 2), indicating that not all 14th peptides of the HA2 protein in different subtype IAVs had a broad-spectrum serum binding activity.

**Fig. 3:**
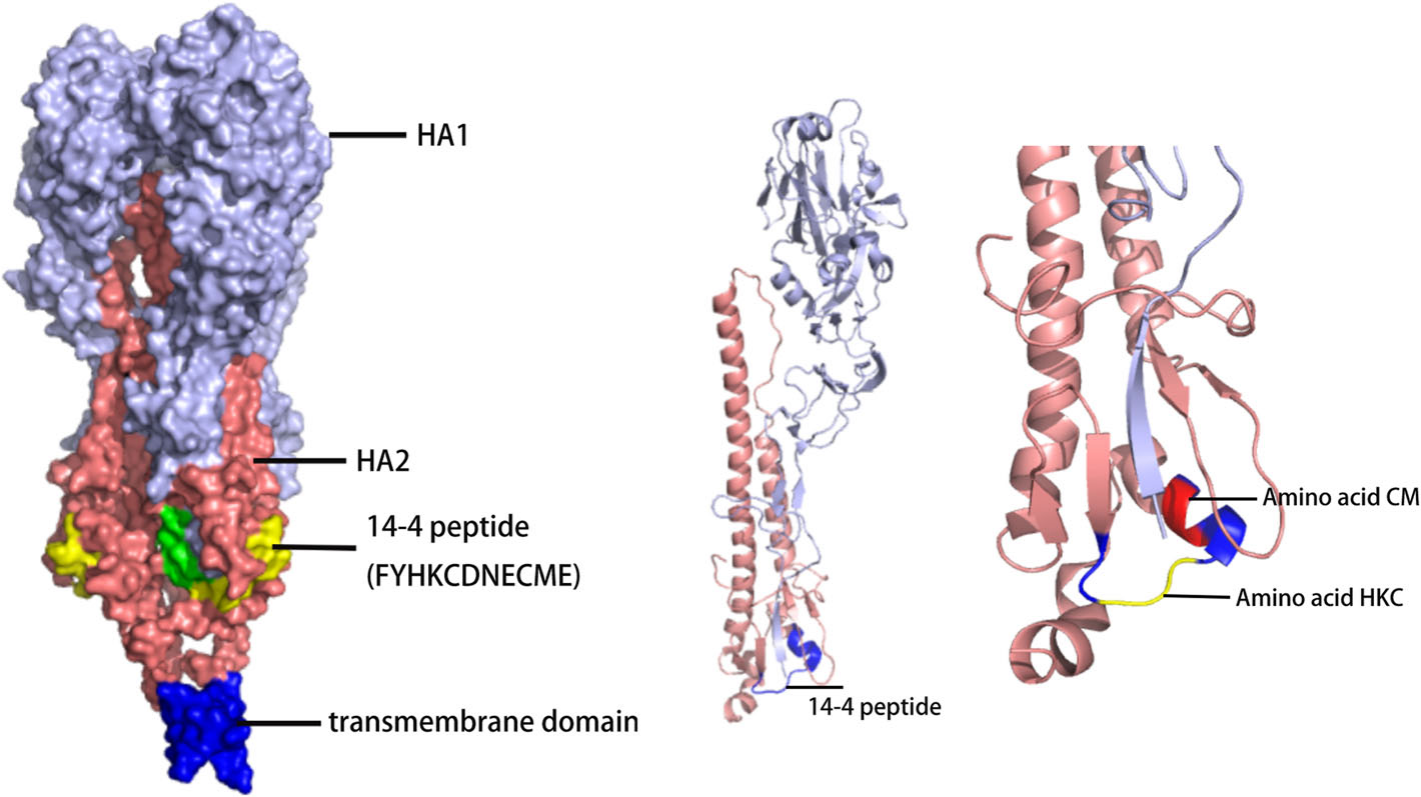
Locations of 14th and 14–4 peptides in simulated HA 3D structure of S strain. The positions of peptide on the hemagglutinin (HA) molecule were analyzed using X-ray crystal structure obtained from the Protein Database. The picture was generated by the SWISS-MODEL system and the PyMOL system

Through PyMOL software and the online protein simulation website SWISS-MODEL, the 3D structure of the HA protein was simulated based on the sequences of S (Fig. 3). The H5 14th peptide consisted of α-helix and loop structures, which was exposed to the surface and able to induce antibody binding.

**Fig. 4:**
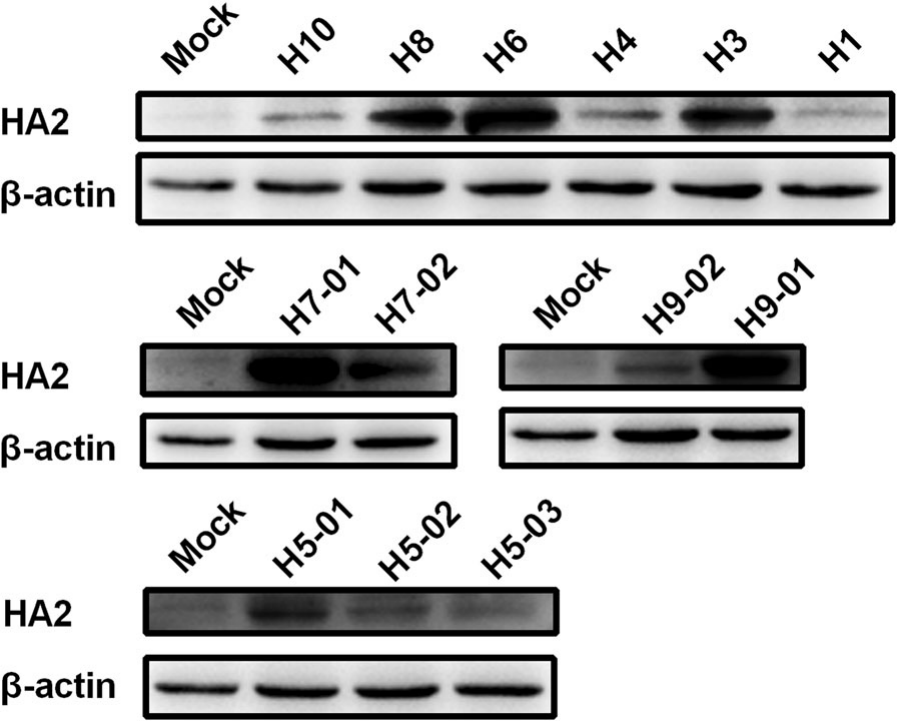
Western-blotting analysis of HA2 protein from different subtypes of influenza A viruses (IAVs). Lysates (amount of protein was 50 μg) of chicken embryo fibroblasts (CEF) infected with IAVs at MOI of 0.01 for 12 h–15 h were incubated with a primary antibody (immune serum of BSA-conjugated H5 14th peptide) and a monoclonal antibody (mAb) against β-actin. Bands were visualized using a chemiluminescence imaging analysis system after incubation with horse radish peroxidase (HRP)-labeled secondary antibodies. H5–01: A/Mallard/Huadong/S/2005; H5–02: A/Duck/Huadong/wx1205/2016; H5–03: A/Goose/Huadong/yz1111/2016; H7–01: A/Chicken/Jiangsu/W1–8/15; H7–02: A/Chicken/Huadong/JD/17; H9–01: A/Chicken/Shanghai/F/98; H9–02: A/Chicken/Taixing/10/2010

To confirm immunogenicity, H5 14th peptide was conjugated to BSA, and used to immunize chickens. Chicken serum was collected for western-blot analysis of different subtypes of IAVs. The results showed that immune serum could bind to HA2 proteins from H1, H3, H4, H5, H6, H7, H8, H9, or H10 subtype IAVs (Fig. 4, Additional file 3), indicating that the H5 14th peptide was as immunogenic as a universal epitope.

### Identification of key amino acids for the universal epitope

To determine specific epitope, H5 14th peptide was further cut into four overlapping peptides and each peptide contained 11 amino acids. The peptides 14–1, 14–2 and 14–3 showed a few positive reactions to sera (Fig. 2a-c, Additional file 2). In contrast, SNR values of the 14–4 peptide serum binding activity were higher than 2 (Fig. 2d, Additional file 2), except for H5–04 serum (equal to 2), indicating that some key amino acids might be in the 14–4 peptide (FYHKCDNECME).

**Fig. 5:**
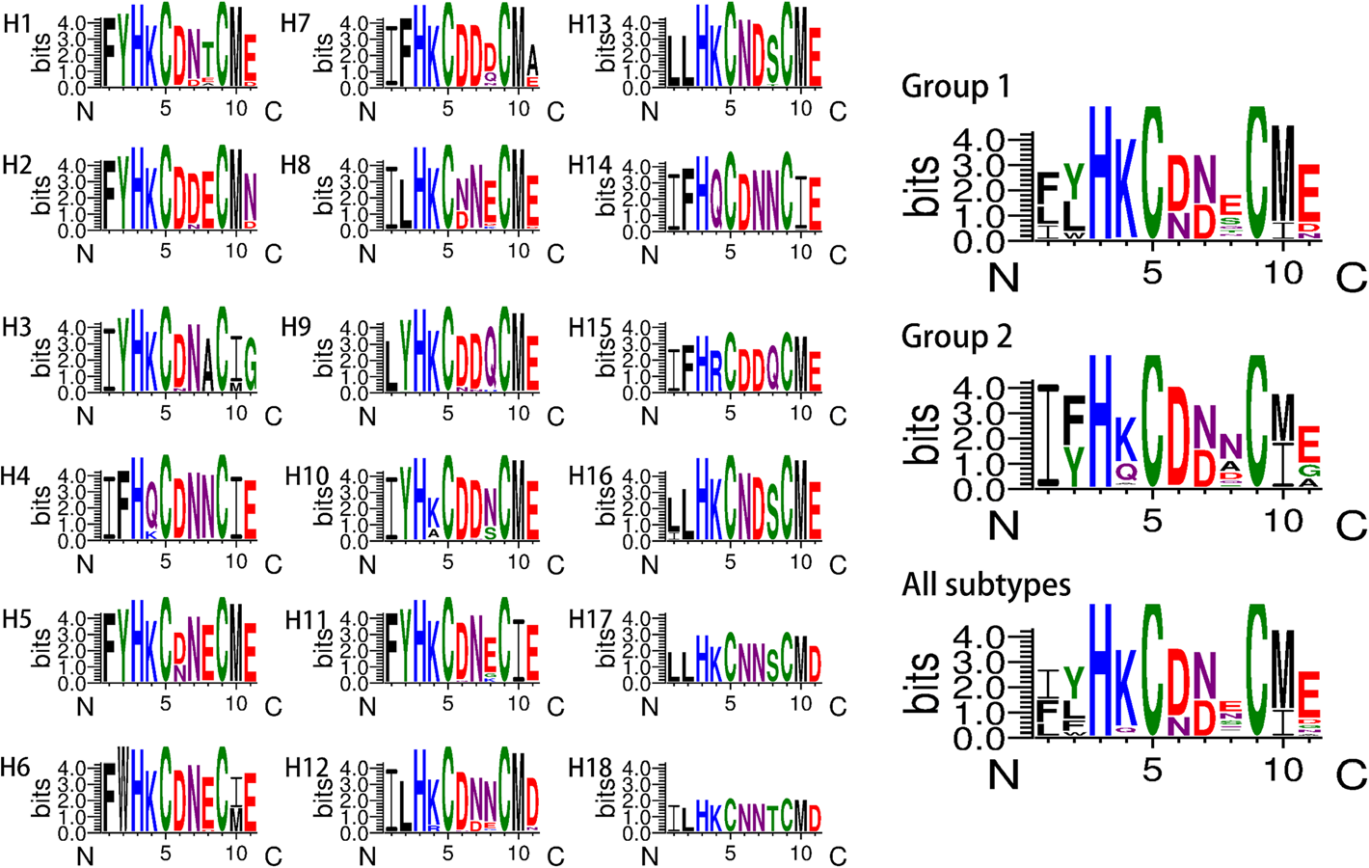
Variation in 14–4 peptide in influenza A viruses was analyzed using WebLogo3. Hemagglutinin (HA) gene sequences of influenza A viruses (IAVs) available from the GISAID and GenBank were aligned by MEGA 7.0 and then analyzed by WebLogo3 (http://weblogo.threeplusone.com/)

**Fig. 6:**
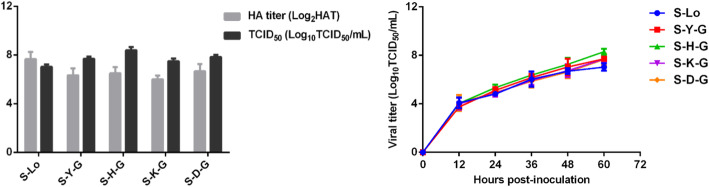
HA titer, median tissue culture infectious dose (TCID_50_) and growth curve of recombinant viruses. Viruses were inoculated into chicken embryo allantoic cavity (9-day-old specific pathogen free chicken embryo, 0.25 μL each) and its Hemagglutination (HA) titer was determined. TCID_50_ was determined through chicken embryo fibroblasts (CEF) and calculated by the Reed–Muench method. To determine growth curve, CEF were infected with each virus at MOI of 0.01 in M199 for 1 h. The infected cells were washed with PBS and then serum-free M199 was added. Cells were incubated at 37 °C under 5% CO2. The virus titers in the supernatant were monitored periodically by determination of TCID_50_ in CEF. Each reaction was repeated for 3 times, and the titers were expressed as means ± standard deviation

**Fig. 7:**
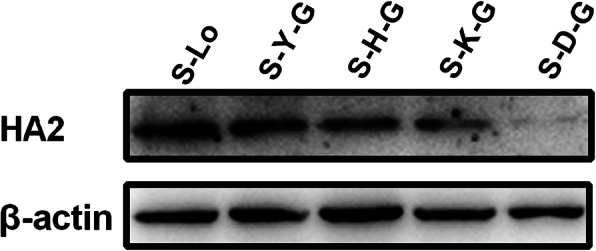
Western-blotting analysis of HA2 protein from mutant viruses. Lysates (amount of protein was 50 μg) of chicken embryo fibroblasts (CEF) infected with mutant viruses at MOI of 0.01 for 12 h–15 h were incubated with a primary antibody against H5 14th peptide and monoclonal antibody (mAb) against β-actin. Bands were visualized by a chemiluminescence imaging analysis system after incubation with horse radish peroxidase (HRP)-labeled secondary antibodies. S-Lo: H5 IAV control strain; S-Y-G: Tyrosine (Y) in 14–4 peptide of H5 IAV was mutated by Glycine (G); S-H-G: Histidine (H) in 14–4 peptide of H5 IAV was mutated by G; S-K-G: Lysine (K) in 14–4 peptide of H5 IAV was mutated by G; S-D-G: Asparticacid (D) in 14–4 peptide of H5 IAV was mutated by G

By comparing sequences and structure of the 14–4 peptide from different subtypes of IAVs (Figs. 3 and 5), a cluster of amino acids (−-HKC---CM-) was conserved in most subtypes of IAVs. However, CM were located at the alpha helix region that was not easy to form epitopes [20]. So, they were not taken into consideration. Based on the sequence of five amino acids (YHKCD), 16 mutation/deletion patterns were designed (Table 1). Among these, all deletion patterns could not be applied to a rescue virus. Only when a single amino acid (Y/H/K/D) was mutated to glycine (G) could the virus be rescued. Rescued viruses were named S-Y-G, S-H-G, S-K-G, and S-D-G (biological characteristics of recombinant viruses are shown in Fig. 6). The binding activities of rescued viruses to H5 14th peptide immune serum were analyzed by western-blotting. The results showed that the band intensities of viruses with substitution of amino acid Y or H to G were similar to that of the wild-type strain. However, the band intensities of viruses with substitutions of the other two amino acids (K or D) to G decreased significantly (Fig. 7, Additional file 3), indicating that amino acids K and D were critical for the serum binding activity of the universal epitope.

**Table 1:** Different mutation/deletion patterns of 14–4 peptide

Mutation	Delete 4 amino acids	Delete 1 amino acid	Delete 5 amino acids
F**G**HKCDNECME	FY----NECME	F-HKCDNECME	F-----NECME
FY**G**KCDNECME	F-H---NECME	FY-KCDNECME	
FYH**G**CDNECME	F--K--NECME	FYH-CDNECME	
FYHK**G**DNECME	F---C-NECME	FYHK-DNECME	
FYHKC**G**NECME	F----DNECME	FYHKC-NECME	

- represents deletion, bold letter represents substitution amino acid

H5 14–4 peptide was analyzed by using IEDB. The results showed that several epitopes containing partial amino acids of 14–4 peptide were confirmed as MHC-II-restricted T cell epitope and had effects on releasing IL-2 or IFN (Table 2). However, their influence was not significant [21–23].

**Table 2:** Results of 14–4 peptide analyzed in IEDB

Epitope	Parent protein	Antigen name	Host	Method	Assay	Result	Reference
CFEFYHKCNNECMESVK	hemagglutinin (480–496)	A/NewCaledonia/20/1999 (H1N1)	*Homo sapiens*	ELISPOT	IFNg release	Positive	Jenny Aurielle B Babon, Hum Immunol, 2009
YHKCNNECMESVKNGTYD	Hemagglutinin (484–501)	A/NewCaledonia/20/1999 (H1N1)	*Mus musculus* HLA-DR1 Tg	ELISPOT	IL-2 release	Positive	Katherine A Richards, Immunology, 2011
EFYHKCDNECMES	hemagglutinin (485–497)	A/Thailand/4(SP-528)/2004 (H5N1)	*Mus musculus* HLA-DR1 Tg	ELISPOT	IL-2 release	Positive	Katherine A Richards, J Virol, 2009
EEMGNGCFKIYHKCD	Hemagglutinin (476–490)	A/X-31 (H3N2)	*Mus musculus* C57BL/6	ELISPOT	IFNg release	Negative	Sherry R Crowe, Vaccine, 2006
FEFYHKCDNECMESV	Hemagglutinin (481–495)	A/PuertoRico/8/34/Mount Sinai (H1N1)	*Mus musculus *C57BL/6	ELISPOT	IFNg release	Negative	Sherry R Crowe, Vaccine, 2006
IGNGCFEFYHKCDNE	Hemagglutinin (476–490)	A/PuertoRico/8/34/MountSinai (H1N1)	*Mus musculus* C57BL/6	ELISPOT	IFNg release	Negative	Sherry R Crowe, Vaccine, 2006
AEDMGNGCFKIYHKCDN	Hemagglutinin (475–491)	A/New York/384/2005 (H3N2)	*Homo sapiens*	ELISPOT	IFNg release	Negative	Jenny Aurielle B Babon, Hum Immunol, 2009
AKELGNGCFEFYHKCDN	Hemagglutinin (476–492)	A/Thailand/4(SP-528)/2004 (H5N1)	*Homo sapiens*	ELISPOT	IFNg release	Negative	Jenny Aurielle B Babon, Hum Immunol, 2009
GCFEFYHKCDNECMESV	Hemagglutinin (482–498)	A/Thailand/4(SP-528)/2004 (H5N1)	*Homo sapiens*	ELISPOT	IFNg release	Negative	Jenny Aurielle B Babon, Hum Immunol, 2009
HKCDNECMESVRNGTYD	Hemagglutinin (488–504)	A/Thailand/4(SP-528)/2004 (H5N1)	*Homo sapiens*	ELISPOT	IFNg release	Negative	Jenny Aurielle B Babon, Hum Immunol, 2009
GNGCFEFYHKCNNECMES	Hemagglutinin (477–494)	A/NewCaledonia/20/1999 (H1N1)	*Mus musculus* HLA-DR1 Tg	ELISPOT	IL-2 release	Negative	Katherine A Richards, J Virol, 2007

## Discussion

Owing to natural and immune selection pressures, IAVs constantly evolve by antigenic drift or antigenic shift, resulting in influenza epidemics and recurring pandemics with serious consequences for public health and animal production [24]. Although monitoring IAV mutations to update vaccine strains is the primary method to achieve a suitable vaccine, increasingly more effort is put on finding a universal vaccine to prevent and control influenza epidemics [25–29].

The neutralizing antibody induced by the HA1 protein of IAV has a strong protective effect; however, it is well known that HA1 is highly variable. Although HA stalk based universal vaccine provides protection against group 2 IAVs [30, 31], HA2 with high sequence and structure conservation among various subtypes of IAVs are more suitable as targets for screening broad-spectrum epitopes [32]. It has been proved that HA2 has universal epitopes crossing Group 1 and Group 2 IAVs [16, 17, 33], which are usually screened by effective monoclonal antibodies and whole viruses or peptides. In this study, we applied peptide microarray with immune serum against different subtypes of IAVs to screen a universal antigenic epitope.

**Fig. 8:**
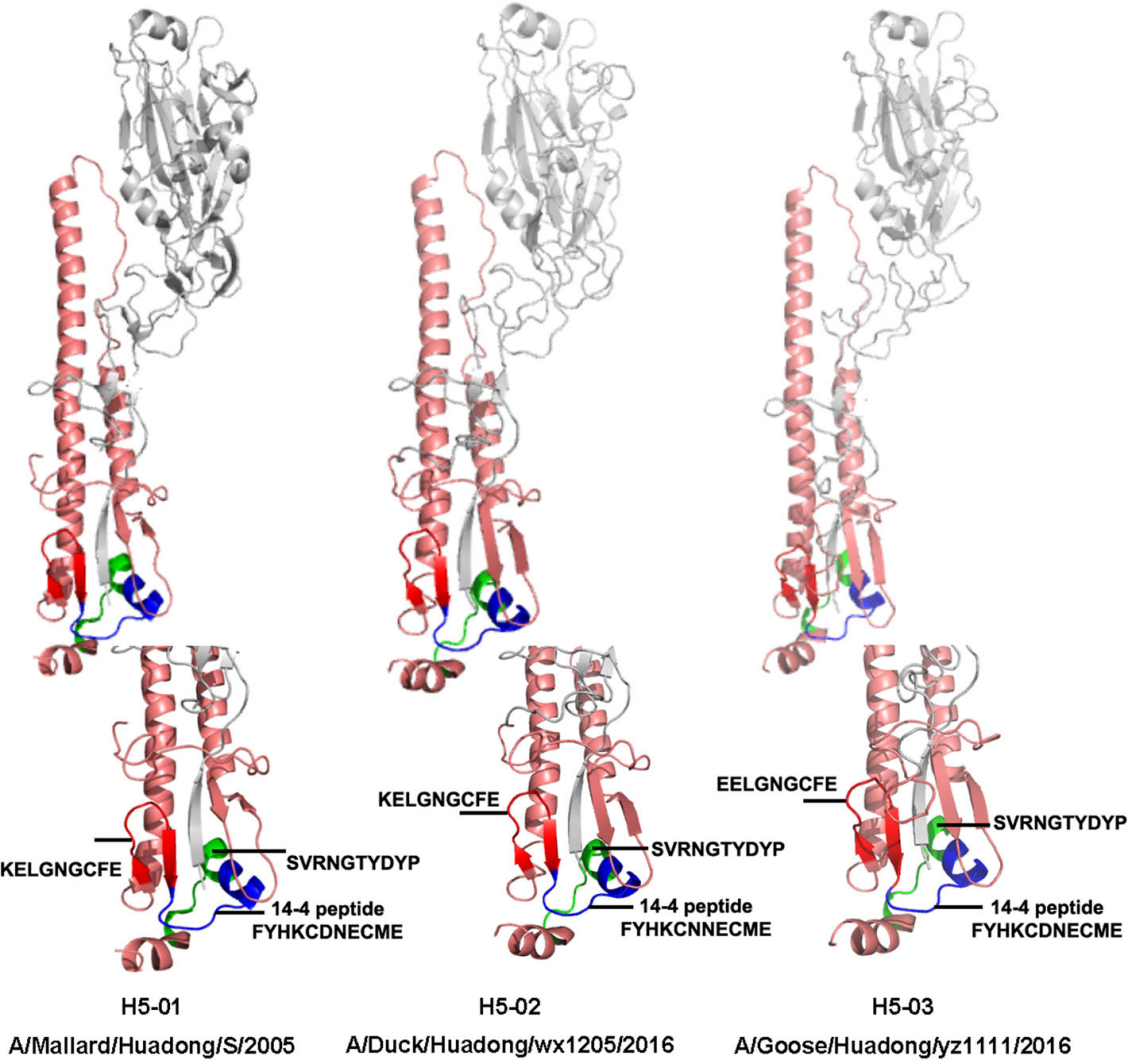
HA 3D structures of H5–01, H5–02 and H5–03. According to PDB ID 4JUK, 6NTF, and 6PCX, the HA structures of H5–01 (A/Mallard/Huadong/S/2005), H5–02 (A/Duck/Huadong/wx1205/2016), and H5–03 (A/Goose/Huadong/yz1111/2016) were simulated through SWISS-MODEL system and analyzed by PyMOL system

The peptide microarray is a new type of technology that has recently been developed [34–36]. In a dot matrix, proteins are decomposed into a plurality of peptides and dotted on silica gel film [34, 35]. It can not only detect the corresponding antibody in serum but also reverse deduce the epitope recognized by the antibody [36, 37]. Based on the peptide microarray, we screened out the H5 14th peptide, KELGNGCFEFYHKCDNECME in HA2, which was positively responsive to the antibodies against multiple subtypes of IAVs. Furthermore, the truncated peptide (FYHKCDNECME) was confirmed to play a major role in the functioning of the broad-spectrum serum binding activity. In the mimetic 3D protein structure, the peptide was located at the bottom of the HA stem region, while the truncated peptide was located outside the bottom of the peptide, suggesting that the peptide epitope was exposed and immunogenic. In fact, the H5 14th peptide showed well binding activity against sera collected from IAVs infected chickens (Data not shown). However, it’s confusing that the reactivity of the three different HA2 from the H5–01, H5–02 and H5–03 viruses against a serum specific of H5 14th peptide was different (Fig. 4). So, HA 3D simulation structures of those viruses were also simulated. According to Table 3 and Fig. 8, the H5 14th peptide sequences and structures of three viruses are not significantly different, but different protein structures around them may affect the reaction of the antibody with the epitope.

**Table 3:** Amino acid sequences of 14th peptide and 14–4 peptide of 13 virus

Virus	Type	14th peptide	14–4 peptide
A/Duck/Eastern China/103/2003	H1N1	KEIGNGCFEFYHKCNNECME	FYHKCNNECME
A/Duck/Eastern China/852/2003	H3N2	EDMGNGCFKIYHKCDNACIE	IYHKCDNACIE
A/Duck/Eastern China/160/2002	H4N6	EDKGNGCFEIFHQCDNNCIE	IFHQCDNNCIE
A/Mallard/Huadong/S/2005	H5N1	KELGNGCFEFYHKCDNECME	FYHKCDNECME
A/Duck/Huadong/wx1205/2016	H5N1	KELGNGCFEFYHKCNNECME	FYHKCNNECME
A/Goose/Huadong/yz1111/2016	H5N6	EELGNGCFEFYHKCDNECME	FYHKCDNECME
A/Duck/Eastern China/58/2003	H6N2	NDLGNGCFEFWHKCDNECIE	FWHKCDNECIE
A/Chicken/Jiangsu/W1–8/15	H7N9	EEDGTGCFEIFHKCDDDCMA	IFHKCDDDCMA
A/Chicken/Huadong/JD/17	H7N9	EEDGTGCFEIFHKCDDDCMA	FHKCDDDCMA
A/Duck/Eastern China/01/2005	H8N4	IDAGNGCFDILHKCDNECME	ILHKCDNECME
A/Chicken/Shanghai/F/98	H9N2	MEDGKGCFELYHKCDDQCME	LYHKCDDQCME
A/Chicken/Taixing/10/2010	H9N2	MEDGKGCFELYHKCDNQCME	LYHKCDNQCME
A/Chicken/Huadong/RD5/2013	H10N9	EEDGKGCFEIYHKCDDNCME	IYHKCDDNCME

As three consecutive amino acids (HKC) were relatively conserved in the H5 14th peptide of most IAVs, a total of 16 mutation/deletion patterns of the peptide was designed to find key amino acids for binding activity based on consecutive amino acids (YHKCD). Viruses could only be successfully rescued when a single amino acid was mutated to a non-polar amino acid G, except amino acid C. This may demonstrate that the H5 14th peptide structure is an extremely stable conformation in the HA stem region, and any amino acid deletion of the peptide might have a strong effect on stem structure. Interestingly, amino acid C, which is known as the only one kind of amino acid to form intermolecular disulfide bonds, was almost completely conserved in different subtypes of IAVs. In 3D simulation (Fig. 8), 14–4 peptide is also adjacent to HA1. Substitution of C to G failed to rescue the virus, indicating the C was of great significance for stabilizing protein/peptide structures [38–40]. Simultaneously, when “K/D” was mutated to G respectively, the binding activities of viruses to immune serum of the H5 14th peptide decreased. Although both the H5 14th and the H7–14 peptide had the core amino acid HKCD, the former showed more broad-spectrum serum binding activity than that of the H7–14 peptide. These data suggest that the broad-spectrum epitope consists of HKCD and adjacent amino acids.

Additionally, Guo et al also found that the peptide FYHKCDNT was an immunodominant epitope in the 2009 pandemic H1N1 IAV, and the seasonal influenza viruses, induced generation of high volumes of antibodies by organisms [41]. Wang et al proved that peptide CFEFYHKCDNTCMES could be recognized by the antiserum against the H1 subtype swine influenza virus, and was able to generate antibody responses in pigs via intranasal inoculation [42]. Katherine et al identified that peptide YHKCNNECMESVKNGTYD and EFYHKCNNECMES played a role in CD4 T cells response and facilitated the release of IL-2. However, the ELISPOT results of the two peptides only detected less than 50 IL-2-producing cells per million CD4 T cells [22, 23].

This study had two potential limitations. One of them was that nine sera against different subtype IAVs were used for epitope screening, the broad-spectrum of H5 14th peptide should be further confirmed by sera against other subtypes IAVs. Another limitation was that H5 14th peptide for universal vaccine design should be confirmed by animal protection study.

## Conclusion

A peptide FYHKCDNECME was identified demonstrating broad-spectrum serum binding activity to different subtypes of IAVs may be used as a novel universal target for detection and vaccine design.

## Methods

### Virus, serum, plasmid, and cells

A total of 13 strains of IAV (Table 4) isolated from live poultry markets, including nine different subtypes of IAV (H1, H3, H4, H5, H6, H8, H7, H9, and H10), were used to prepare sera. All viruses were propagated in specific pathogen free (SPF) embryonated chicken eggs. Two kinds of sera against the H5 subtype of vaccine strains (2.3.4.4d and 2.3.2.1d) were purchased from YEBIO Company (China). The H5N1 avian influenza virus origin rescue plasmids (pHW-PB2, pHW-PB1, pHW-PA, pHW-HALo, pHW-NP, pHW-NA, pHW-M, and pHW-NS) were constructed by Shi et al [43]. Primary chicken embryo fibroblasts (CEF) were prepared from 9 to 10 d SPF chicken embryos and cultured in M199 (HyClone Laboratories, USA) containing 4% fetal bovine serum (FBS, Shuangru Biotech, China).

**Table 4:** Background information of 13 influenza A viruses (IAVs) and their immunized antisera

Virus	Subtype	HI titer of antisera (nlog_2_ ± SD)
A/Duck/Eastern China/103/2003	H1N1	7.5 ± 0.7
A/Duck/Eastern China/852/2003	H3N2	7.3 ± 0.5
A/Duck/Eastern China/160/2002	H4N6	6.5 ± 1.1
A/Mallard/Huadong/S/2005	H5N1	8.2 ± 0.2
A/Duck/Huadong/wx1205/2016	H5N1	8.0 ± 0.0
A/Goose/Huadong/yz1111/2016	H5N6	8.0 ± 0.4
A/Duck/Eastern China/58/2003	H6N2	7.3 ± 0.5
A/Chicken/Jiangsu/W1–8/15	H7N9	7.7 ± 1.2
A/Chicken/Huadong/JD/17	H7N9	8.3 ± 0.6
A/Duck/Eastern China/01/2005	H8N4	8.0 ± 0.4
A/Chicken/Shanghai/F/98	H9N2	9.0 ± 0.4
A/Chicken/Taixing/10/2010	H9N2	9.2 ± 0.2
A/Chicken/Huadong/RD5/2013	H10N9	7.5 ± 0.7

### Serum preparation

Forty-five 21-day-old SPF chickens (Beijing Meria Vitong experimental animal technology co., Ltd., China) were housed in cages under biosafety conditions with ad libitum access to food and water, three chickens in each group were immunized with inactivated IAVs (10^6^ EID_50_) with oil emulsion adjuvant or BSA-conjugated peptides (GL Biochem Ltd., China) with Freund’s adjuvant (Sigma, USA) and boosted at a two–week interval. Chickens were inoculated with PBS as control group. Chickens were euthanatized by manual cervical dislocation at 2 weeks after the second vaccination and their sera were collected and identified by hemagglutination inhibition assay for whole virus immunized antisera (Table 4), and peptide chip for peptide immunized antisera.

### Microarray experiment

According to the deduced amino acid sequence of the HA2 protein of the H5 subtype virus A/Mallard/Huadong/S/2005 (S, GenBank accession numbers: EU195389-EU195396), overlapping peptides (10 amino acids overlapped between two adjacent peptides) were synthesized by GL Biochem Ltd. (China), except for the failure of the 11th peptide. To confirm key amino acids for the serum binding activity, the selected H5 14th peptide from the HA2 protein (KELGNGCFEFYHKCDNECME) was further cut into four overlapping peptides. To verify whether the selected peptide from the other subtype virus had similar serum binding activity, the H7–14 peptide from the HA2 protein (A/Chicken/Huadong/JD/17) was synthesized (Table 5). Synthetic peptides were sampled onto iPDMS (modified silica gel film) and workflow of the microarray was mainly based on the previous study [44]. Sera were diluted 1:50 (v/v) with serum dilution buffer (GuardianTM Peroxidase Conjugate Stabilizer/Diluent, Thermo Fisher Scientific, USA) and a 200 μL dilution was added in each well of the chip. The chip was incubated on a shaker for 30 min (500 r/min, 37 °C) and subsequently washed three times with TBST (20 mM Tris-base, pH 6.8, 137 mM NaCl, 0.1% Tween 20). Following incubation with 100 μL of 1:10000 diluted HRP (horseradish peroxidase)-labeled goat anti-chicken IgY for an additional 30 min and washing three times with TBST, 15 μL chemiluminescent substrate (SuperSignal West Pico PLUS Chemiluminescent Substrate, Thermo Fisher, USA) was added to each well of the chip. Chemiluminescent signals were captured by a CCD (charge coupled device) camera (LAS4000 imaging system, GE Healthcare Life Sciences, USA) and saved as an image in TIFF format. Thereafter, chemiluminescence intensity of each peptide point and background was converted into the signal-to-noise ratio (SNR, SNR = (signal strength–background intensity)/background intensity) using GenePix Pro 6.0 software. SNR ≥2 were identified as seropositive [36].

**Table 5:** Amino acid sequence of synthesized peptides

Peptide name	Amino acid
1st	GLFGAIAGFIEGGWQGMVDG
2nd	EGGWQGMVDGWYGYHHSNEQ
3rd	WYGYHHSNEQGSGYAADKES
4th	GSGYAADKESTQKAIDGVTN
5th	TQKAIDGVTNKVNSIIDKMN
6th	KVNSIIDKMNTQFEAVGREF
7th	TQFEAVGREFNNLERRIENL
8th	NNLERRIENLNKKMEDGFLD
9th	NKKMEDGFLDVWTYNAELLV
10th	VWTYNAELLVLMENERTLDF
12th	HDSNVKNLYDKVRLQLRDNA
13th	KVRLQLRDNAKELGNGCFEF
14th	KELGNGCFEFYHKCDNECME
15th	YHKCDNECMESVRNGTYDYP
16th	SVRNGTYDYPQYSEEARLKR
17th	QYSEEARLKREEISGVKLES
18th	EEISGVKLESIGTYQILSIY
19th	IGTYQILSIYSTVASSLALA
H7–14	EEDGTGCFEIFHKCDDDCMA
14–1	KELGNGCFEFY
14–2	GNGCFEFYHKC
14–3	CFEFYHKCDNE
14–4	FYHKCDNECME

### Site-direct mutagenesis and virus rescue

Site-direct mutagenesis of selected amino acid residues on the S strain HA2 protein was performed by the Mut Express II Fast Mutagenesis Kit V2 (Vazyme Biotech, China). Modified HA genes were inserted into the pHW2000 vector [45] and confirmed by sequencing (BGI Company, China). Recombinant viruses were rescued via an 8-plasmid reverse genetics technology as described previously [46]. HEC293T and M90 cells were plated at a ratio of approximately 1.5:1 in six-well plates and cultured in Dulbecco’s Modified Eagle Medium (DMEM) medium (HyClone Laboratories, USA) containing 10% FBS. The modified HA plasmid combined with seven S strain rescue plasmids were transfected using the PolyJet™ transfection reagent (SignaGen Laboratories, USA). At 48–72 h post-transfection, the cells and supernatant were collected and inoculated into chicken embryo allantoic cavity (7-day-old SPF chicken embryo, 0.25 μL each) to propagate recombinant viruses. The Median Tissue Culture Infectious Dose (TCID_50_) of rescued viruses in CEF were determined according to Wagner (2000) and calculated by the Reed–Muench method [47]. To determine growth curve, CEF were infected with each virus at MOI of 0.01 in M199 for 1 h. The infected cells were washed with PBS and then serum-free M199 was added. Cells were incubated at 37 °C under 5% CO2. The virus titers in the supernatant were monitored periodically by determination of TCID_50_ in CEF.

### Western-blot analysis

Chicken embryo fibroblasts was inoculated with viruses (MOI = 0.01) and incubated for 1 h at 37 °C. Cells were washed twice with phosphate buffered saline (PBS, pH 7.2). Thereafter, M199 medium containing 1% FBS was added and incubated for 12 h. Cells were washed once with pre-cooled PBS (4 °C) and lysed with 200 μL of RIPA Lysis Buffer (strong) (CWBIO, Beijing, China) individually on ice for 15–20 min. Supernatants were collected by centrifugation at 12000 r/min for 10 min at 4 °C and mixed with protein loading buffer (Beyotime Biotechnology, China). Following boiling at 100 °C for 6–8 min, samples were subjected to 12% SDS-PAGE, and transferred to a PVDF membrane. The membrane which was first blocked in TBST containing 5% non-fat powdered milk at 25 °C for 1 h was incubated with the primary antibody against the H5 14th peptide (diluted to 1:1000 with TBST), and then incubated with the secondary antibody (Goat Anti-Chicken IgY (H + L) HRP, Abcam, USA, diluted 1:5000 with TBST). Meanwhile, protein bands of β-actin were incubated successively with the primary antibody (Anti-β-actin monoclonal antibody, Sigma Company, USA) and secondary antibody (HRP labelled anti-mouse IgG goat polyclonal antibody, Abcam, USA). Protein bands were developed using a chemiluminescence imaging analysis system (Tanon 5200, Tanon Biotech, China).

### Bioinformatics analysis

HA gene sequences of IAVs available from the GISAID (https://platform.gisaid.org/epi3/frontend) and GenBank influenza database (https://www.ncbi.nlm.nih.gov/genomes/FLU/Database/nph-select.cgi#mainform) were aligned by MEGA 7.0 and then analyzed by WebLogo3 (http://weblogo.threeplusone.com/). HA molecule was analyzed by using Protein Data Bank (PDB), SWISS-MODEL system and the PyMOL System (https://pymol.org/2/). Taking PDB file (PDB ID 4JUK, 6NTF, 6PCX) as a template, the amino acid sequence of target virus was modeled by Alignment Mode on SWISS-MODEL. The PDB file of HA protein of target virus was further modified with PyMOL. The epitope was also analyzed via the Immune Epitope Database (IEDB).

## Supplementary Information

**Additional file 1: Figure S1-S4.** The signal-to-noise ratios (SNRs) of binding activity between sera from different subtype influenza A viruses (IAVs) and the other H5 16 peptides (including 1th (A), 2th (B), 3th (C), 4th (D), 5th (E), 6th (F), 7th (G), 8th (H), 9th (I), 10th (J), 12th (K), 13th (L), 16th (M), 17th (N), 18th (O), or 19th (P) peptide). Synthetic peptides were sampled onto iPDMS (modified silica gel film) to form the chip. For the binding activity assay, sera were diluted 1:50 (v/v) with serum dilution buffer. Signal-to-noise ratio (SNR, SNR = signal strength–background intensity)/background intensity) was determined by GenePix Pro 6.0 software. The dotted line represents the SNR = 2. H5–01: serum against A/Mallard/Huadong/S/2005; H5–02: serum against A/Duck/Huadong/wx1205/2016; H5–03: serum against A/Chicken/Huadong/yz1111/2016; H5–04: serum against 2.3.4.4d vaccine strain; H5–05: serum against 2.3.2.1d vaccine strain; H7–01: serum against A/Chicken/Jiangsu/W1–8/15; H7–02: serum against A/Chicken/Huadong/JD/17; H9–01: serum against A/Chicken/Shanghai/F/98; H9–02: serum against A/Chicken/Taixing/10/2010. Each reaction was repeated for 3 times, SNR values were expressed as means ± standard deviation. X axis, sera against different subtypes of IAVs; Y axis, SNR values.

**Additional file 2:.** Microarray data. Synthetic peptides were sampled onto iPDMS (modified silica gel film) to form the chip. For the binding activity assay, sera were diluted 1:50 (v/v) with serum dilution buffer. Signal-to-noise ratio (SNR, SNR = signal strength–background intensity)/background intensity) was determined by GenePix Pro 6.0 software. H5–01: serum against A/Mallard/Huadong/S/2005; H5–02: serum against A/Duck/Huadong/wx1205/2016; H5–03: serum against A/Chicken/Huadong/yz1111/2016; H5–04: serum against 2.3.4.4d vaccine strain; H5–05: serum against 2.3.2.1d vaccine strain; H7–01: serum against A/Chicken/Jiangsu/W1–8/15; H7–02: serum against A/Chicken/Huadong/JD/17; H9–01: serum against A/Chicken/Shanghai/F/98; H9–02: serum against A/Chicken/Taixing/10/2010. Each reaction was repeated for 3 times, and SNRs were expressed as means ± standard deviation.

**Additional file 3:.** Original images for Western-blotting analysis. Western-blotting analysis of HA2 protein from different subtypes of influenza A viruses (Fig. 4) and mutant viruses (Fig. 7).

## Data Availability

All data generated or analyzed during this study are included in this published article and its supplementary information files.

## Abbreviations

IAV: Influenza a virus
HA: Hemagglutinin
CTL: Cytotoxic t lymphocyte
ELISA: Enzyme linked immunosorbent assay
SPF: Specific pathogen free
CEF: Chicken embryo fibroblasts
FBS: Fetal bovine serum
BSA: Bovine serum albumin
iPDMS: Modified silica gel film
TBST: Tris-buffered saline tween-20
CCD: Charge coupled device
SNR: Signal-to-noise ratio
DMEM: Dulbecco’s modified eagle medium
TCID_50_: Median tissue culture infectious dose
PBS: Phosphate buffered saline
PVDF: Polyvinylidene fluoride
HRP: Horseradish peroxidase
