# Supplementary figures and images for: Identification of a universal antigen epitope of influenza A virus using peptide microarray
Source: BMC Vet Res. 2021 Jan 7;17:22. doi: 10.1186/s12917-020-02725-5 (PMC7792037; doi:10.1186/s12917-020-02725-5)

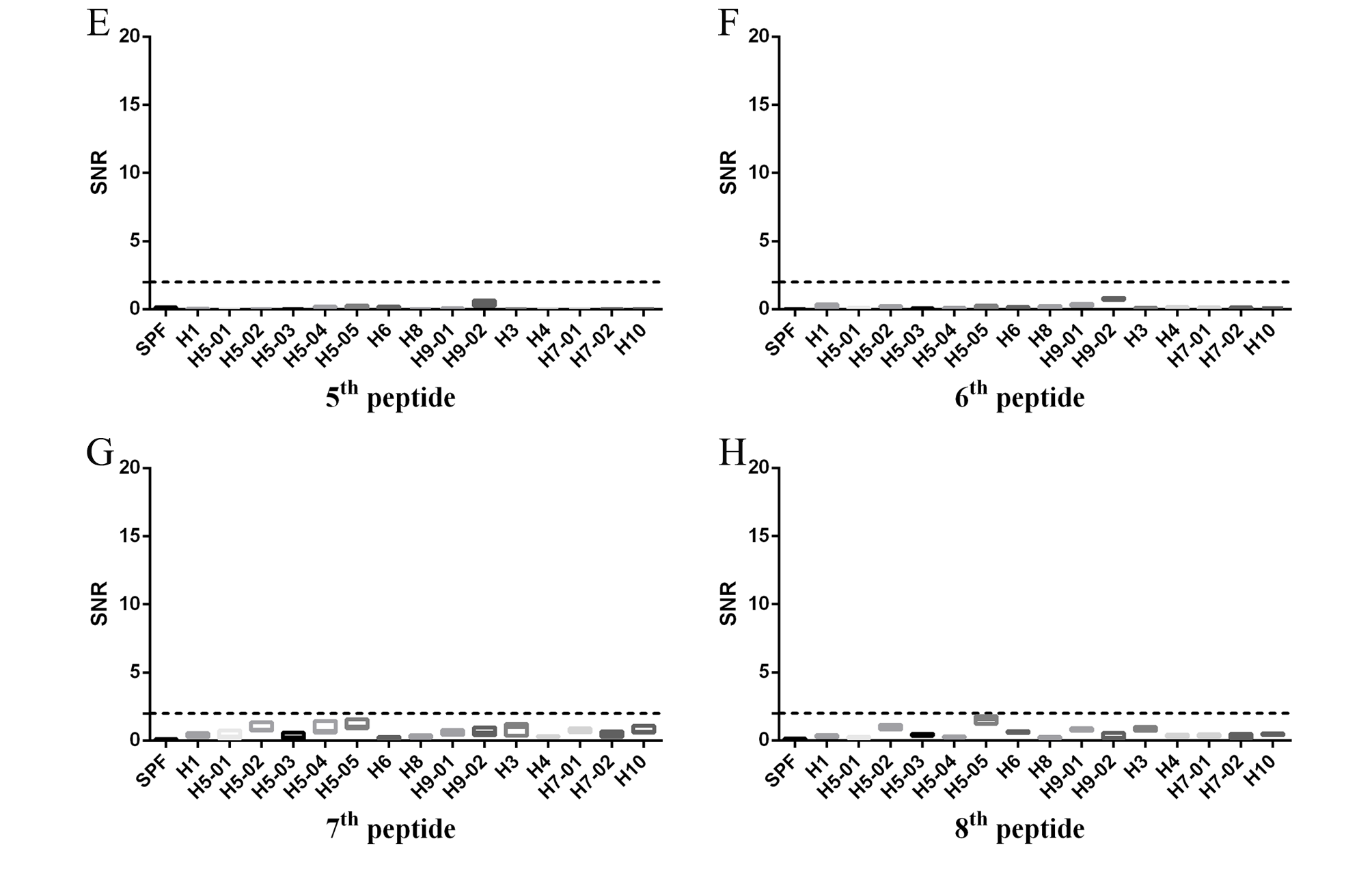

Supplement: Supplementary file 1 — Additional file 1: Figure S1-S4. The signal-to-noise ratios (SNRs) of binding activity between sera from different subtype influenza A viruses (IAVs) and the other H5 16 peptides (including 1th (A), 2th (B), 3th (C), 4th (D), 5th (E), 6th (F), 7th (G), 8th (H), 9th (I), 10th (J), 12th (K), 13th (L), 16th (M), 17th (N), 18th (O), or 19th (P) peptide). Synthetic peptides were sampled onto iPDMS (modified silica gel film) to form the chip. For the binding activity assay, sera were diluted 1:50 (v/v) with serum dilution buffer. Signal-to-noise ratio (SNR, SNR = signal strength–background intensity)/background intensity) was determined by GenePix Pro 6.0 software. The dotted line represents the SNR = 2. H5–01: serum against A/Mallard/Huadong/S/2005; H5–02: serum against A/Duck/Huadong/wx1205/2016; H5–03: serum against A/Chicken/Huadong/yz1111/2016; H5–04: serum against 2.3.4.4d vaccine strain; H5–05: serum against 2.3.2.1d vaccine strain; H7–01: serum against A/Chicken/Jiangsu/W1–8/15; H7–02: serum against A/Chicken/Huadong/JD/17; H9–01: serum against A/Chicken/Shanghai/F/98; H9–02: serum against A/Chicken/Taixing/10/2010. Each reaction was repeated for 3 times, SNR values were expressed as means ± standard deviation. X axis, sera against different subtypes of IAVs; Y axis, SNR values. [file 12917_2020_2725_MOESM1_ESM.zip › renamed_0fed6.tif]

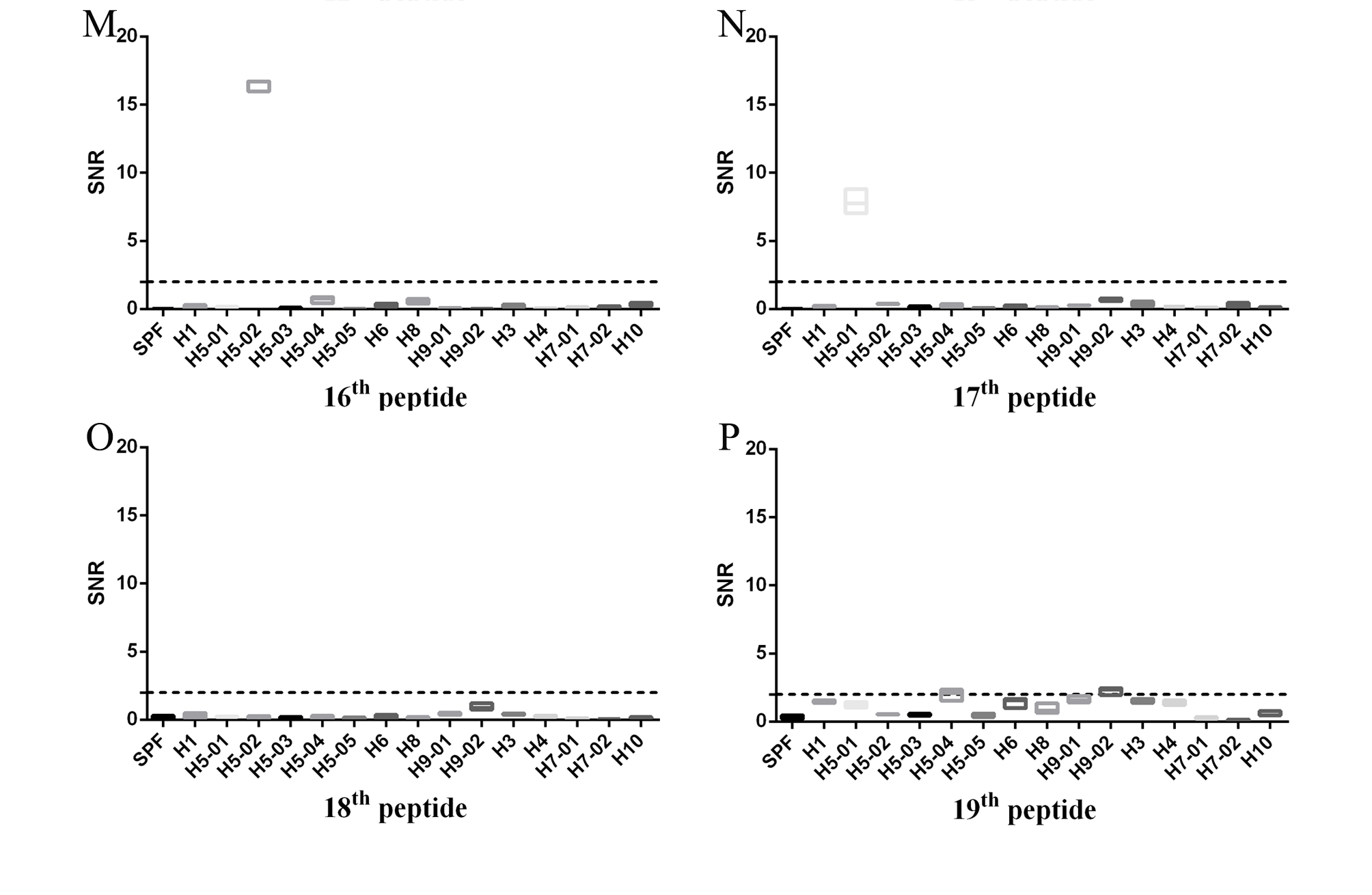

Supplement: Supplementary file 1 — Additional file 1: Figure S1-S4. The signal-to-noise ratios (SNRs) of binding activity between sera from different subtype influenza A viruses (IAVs) and the other H5 16 peptides (including 1th (A), 2th (B), 3th (C), 4th (D), 5th (E), 6th (F), 7th (G), 8th (H), 9th (I), 10th (J), 12th (K), 13th (L), 16th (M), 17th (N), 18th (O), or 19th (P) peptide). Synthetic peptides were sampled onto iPDMS (modified silica gel film) to form the chip. For the binding activity assay, sera were diluted 1:50 (v/v) with serum dilution buffer. Signal-to-noise ratio (SNR, SNR = signal strength–background intensity)/background intensity) was determined by GenePix Pro 6.0 software. The dotted line represents the SNR = 2. H5–01: serum against A/Mallard/Huadong/S/2005; H5–02: serum against A/Duck/Huadong/wx1205/2016; H5–03: serum against A/Chicken/Huadong/yz1111/2016; H5–04: serum against 2.3.4.4d vaccine strain; H5–05: serum against 2.3.2.1d vaccine strain; H7–01: serum against A/Chicken/Jiangsu/W1–8/15; H7–02: serum against A/Chicken/Huadong/JD/17; H9–01: serum against A/Chicken/Shanghai/F/98; H9–02: serum against A/Chicken/Taixing/10/2010. Each reaction was repeated for 3 times, SNR values were expressed as means ± standard deviation. X axis, sera against different subtypes of IAVs; Y axis, SNR values. [file 12917_2020_2725_MOESM1_ESM.zip › renamed_1fe84.tif]

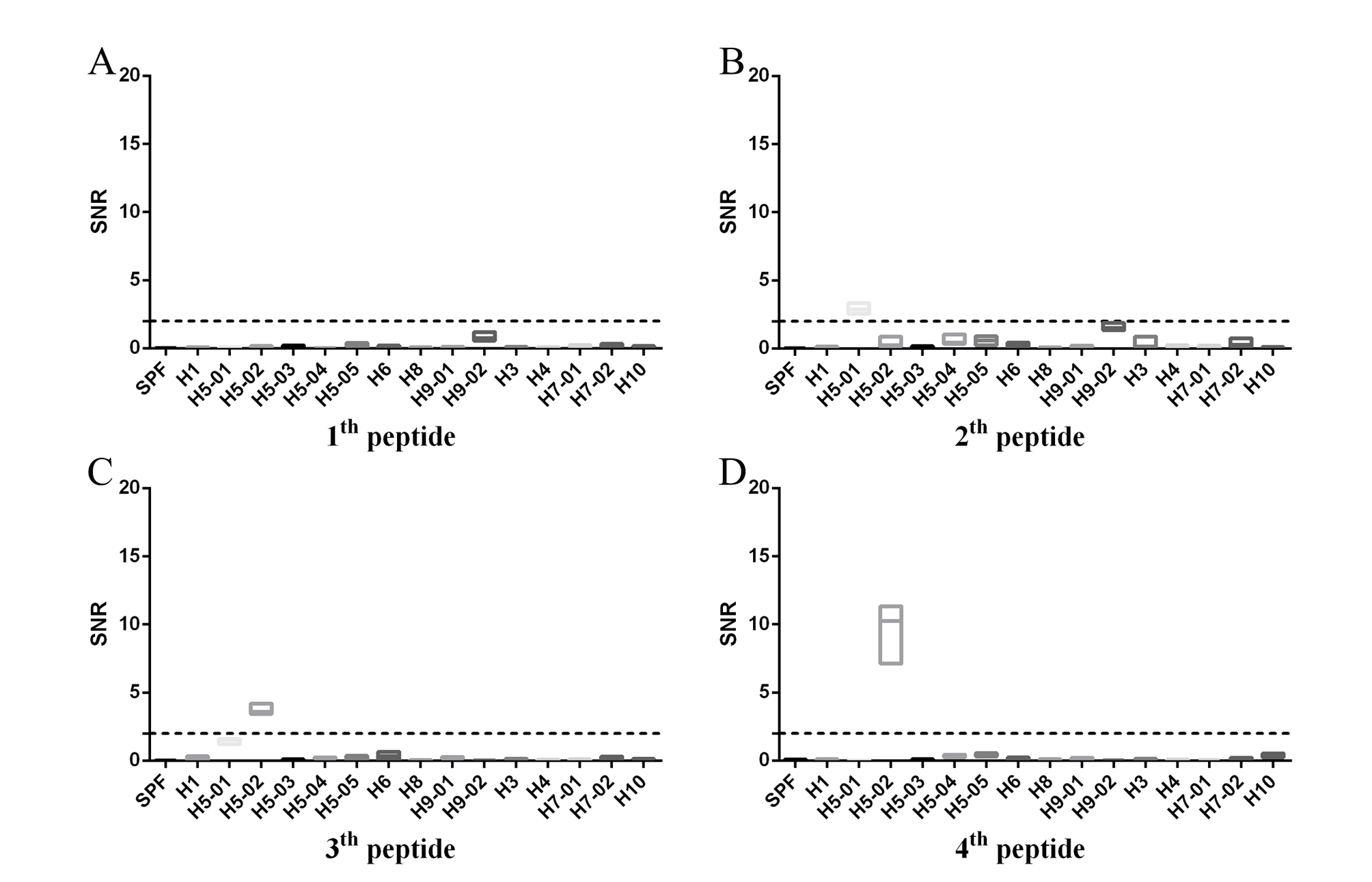

Supplement: Supplementary file 1 — Additional file 1: Figure S1-S4. The signal-to-noise ratios (SNRs) of binding activity between sera from different subtype influenza A viruses (IAVs) and the other H5 16 peptides (including 1th (A), 2th (B), 3th (C), 4th (D), 5th (E), 6th (F), 7th (G), 8th (H), 9th (I), 10th (J), 12th (K), 13th (L), 16th (M), 17th (N), 18th (O), or 19th (P) peptide). Synthetic peptides were sampled onto iPDMS (modified silica gel film) to form the chip. For the binding activity assay, sera were diluted 1:50 (v/v) with serum dilution buffer. Signal-to-noise ratio (SNR, SNR = signal strength–background intensity)/background intensity) was determined by GenePix Pro 6.0 software. The dotted line represents the SNR = 2. H5–01: serum against A/Mallard/Huadong/S/2005; H5–02: serum against A/Duck/Huadong/wx1205/2016; H5–03: serum against A/Chicken/Huadong/yz1111/2016; H5–04: serum against 2.3.4.4d vaccine strain; H5–05: serum against 2.3.2.1d vaccine strain; H7–01: serum against A/Chicken/Jiangsu/W1–8/15; H7–02: serum against A/Chicken/Huadong/JD/17; H9–01: serum against A/Chicken/Shanghai/F/98; H9–02: serum against A/Chicken/Taixing/10/2010. Each reaction was repeated for 3 times, SNR values were expressed as means ± standard deviation. X axis, sera against different subtypes of IAVs; Y axis, SNR values. [file 12917_2020_2725_MOESM1_ESM.zip › renamed_43ce8.tif]

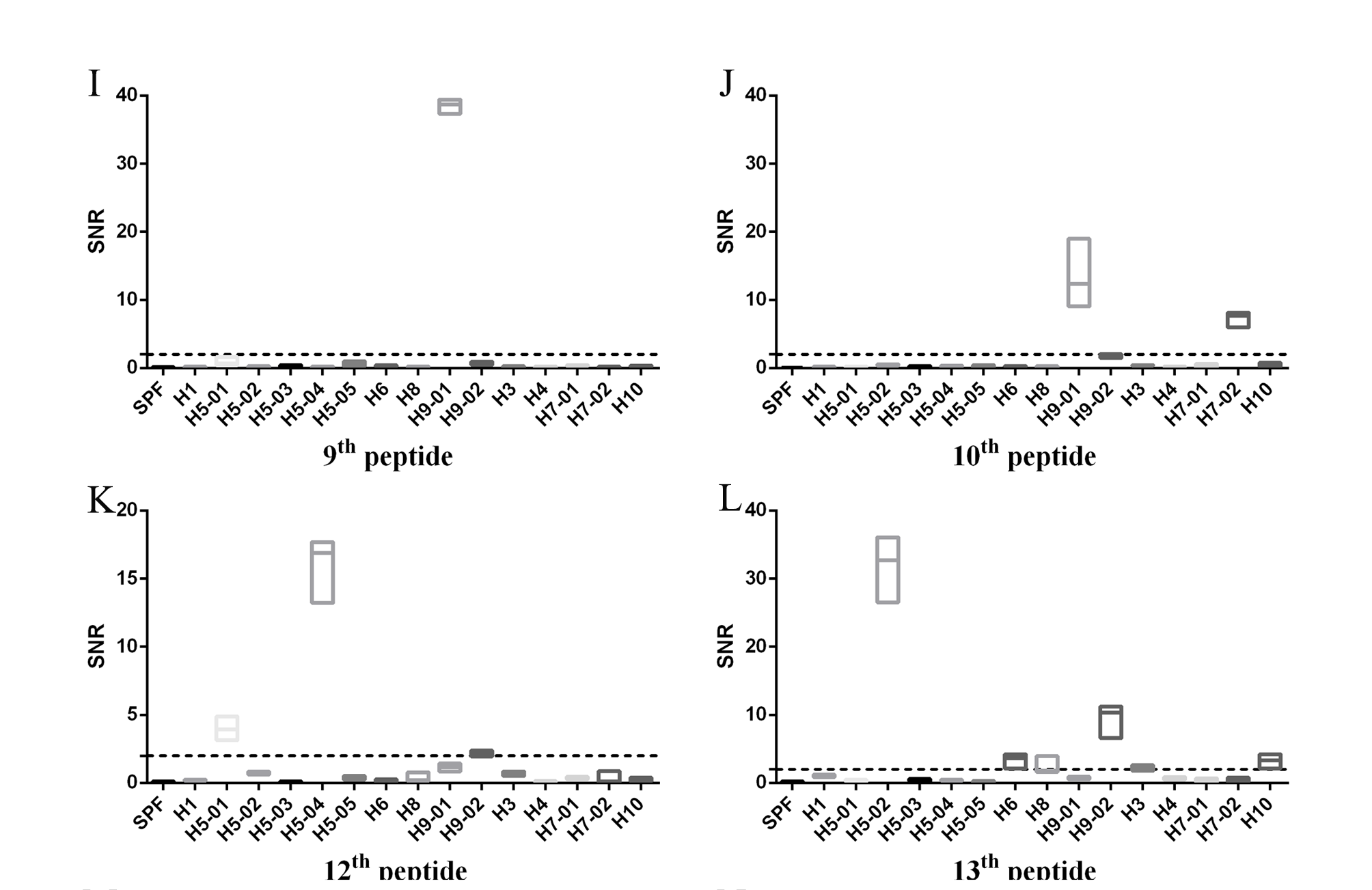

Supplement: Supplementary file 1 — Additional file 1: Figure S1-S4. The signal-to-noise ratios (SNRs) of binding activity between sera from different subtype influenza A viruses (IAVs) and the other H5 16 peptides (including 1th (A), 2th (B), 3th (C), 4th (D), 5th (E), 6th (F), 7th (G), 8th (H), 9th (I), 10th (J), 12th (K), 13th (L), 16th (M), 17th (N), 18th (O), or 19th (P) peptide). Synthetic peptides were sampled onto iPDMS (modified silica gel film) to form the chip. For the binding activity assay, sera were diluted 1:50 (v/v) with serum dilution buffer. Signal-to-noise ratio (SNR, SNR = signal strength–background intensity)/background intensity) was determined by GenePix Pro 6.0 software. The dotted line represents the SNR = 2. H5–01: serum against A/Mallard/Huadong/S/2005; H5–02: serum against A/Duck/Huadong/wx1205/2016; H5–03: serum against A/Chicken/Huadong/yz1111/2016; H5–04: serum against 2.3.4.4d vaccine strain; H5–05: serum against 2.3.2.1d vaccine strain; H7–01: serum against A/Chicken/Jiangsu/W1–8/15; H7–02: serum against A/Chicken/Huadong/JD/17; H9–01: serum against A/Chicken/Shanghai/F/98; H9–02: serum against A/Chicken/Taixing/10/2010. Each reaction was repeated for 3 times, SNR values were expressed as means ± standard deviation. X axis, sera against different subtypes of IAVs; Y axis, SNR values. [file 12917_2020_2725_MOESM1_ESM.zip › renamed_f58ef.tif]
