# Supplementary material for: Identification of a universal antigen epitope of influenza A virus using peptide microarray
Source: BMC Vet Res. 2021 Jan 7;17:22. doi: 10.1186/s12917-020-02725-5 (PMC7792037; doi:10.1186/s12917-020-02725-5)
Supplement: Supplementary file 3 — Additional file 3:. Original images for Western-blotting analysis. Western-blotting analysis of HA2 protein from different subtypes of influenza A viruses (Fig. 4) and mutant viruses (Fig. 7). [file 12917_2020_2725_MOESM3_ESM.docx]

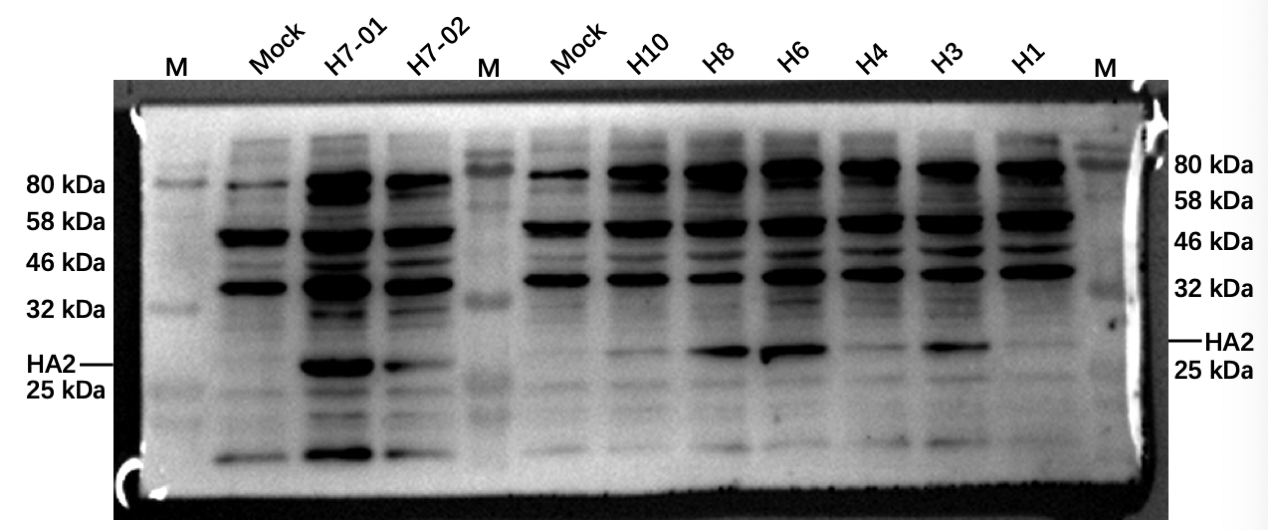


**Fig 1 Original blot of figure 4 in text, Western-blotting analysis of HA2 protein from H1, H3, H4, H6, H7, H8 and H10 subtypes IAVs infected samples:** Lysates of chicken embryo fibroblasts (CEF) infected with IAVs at MOI of 0.01 for 12 h–15 h were incubated with a primary antibody against the 14^th^ peptide. Bands were visualized using a chemiluminescence imaging analysis system after incubation with horse radish peroxidase (HRP)-labeled secondary antibodies. H7-01: A/Chicken/Jiangsu/W1-8/15; H7-02: A/Chicken/Huadong/JD/17


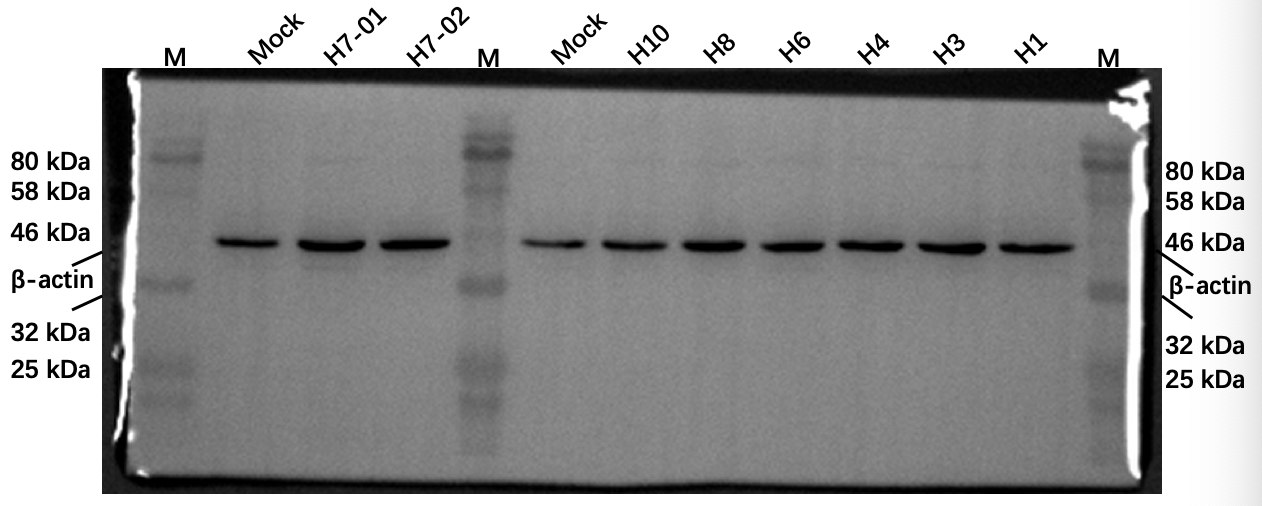


**Fig 2 Original blot of figure 4 in text, Western-blotting analysis of** **β-actin from H1, H3, H4, H6, H7, H8 and H10 subtypes IAVs infected samples:** Lysates of chicken embryo fibroblasts (CEF) infected with IAVs at MOI of 0.01 for 12 h–15 h were incubated with a monoclonal antibody (mAb) against β-actin. Bands were visualized using a chemiluminescence imaging analysis system after incubation with horse radish peroxidase (HRP)-labeled secondary antibodies. H7-01: A/Chicken/Jiangsu/W1-8/15; H7-02: A/Chicken/Huadong/JD/17


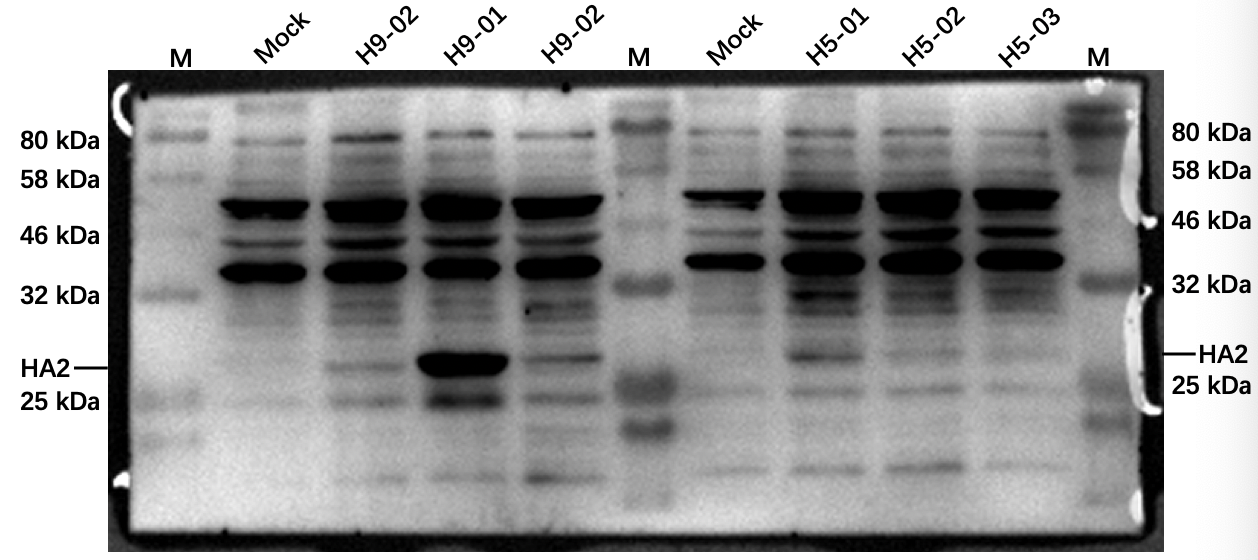


**Fig 3 Original blot of figure 4 in text, Western-blotting analysis of HA2 protein from H5 and H9 subtypes IAVs infected samples:** Lysates of chicken embryo fibroblasts (CEF) infected with IAVs at MOI of 0.01 for 12 h–15 h were incubated with a primary antibody against the 14^th^ peptide. Bands were visualized using a chemiluminescence imaging analysis system after incubation with horse radish peroxidase (HRP)-labeled secondary antibodies. H9-01: A/Chicken/Shanghai/F/98; H9-02: A/Chicken/Taixing/10/2010; H5-01: A/Mallard/Huadong/S/2005; H5-02: A/Duck/Huadong/wx1205/2016; H5-03: A/Goose/Huadong/yz1111/2016.


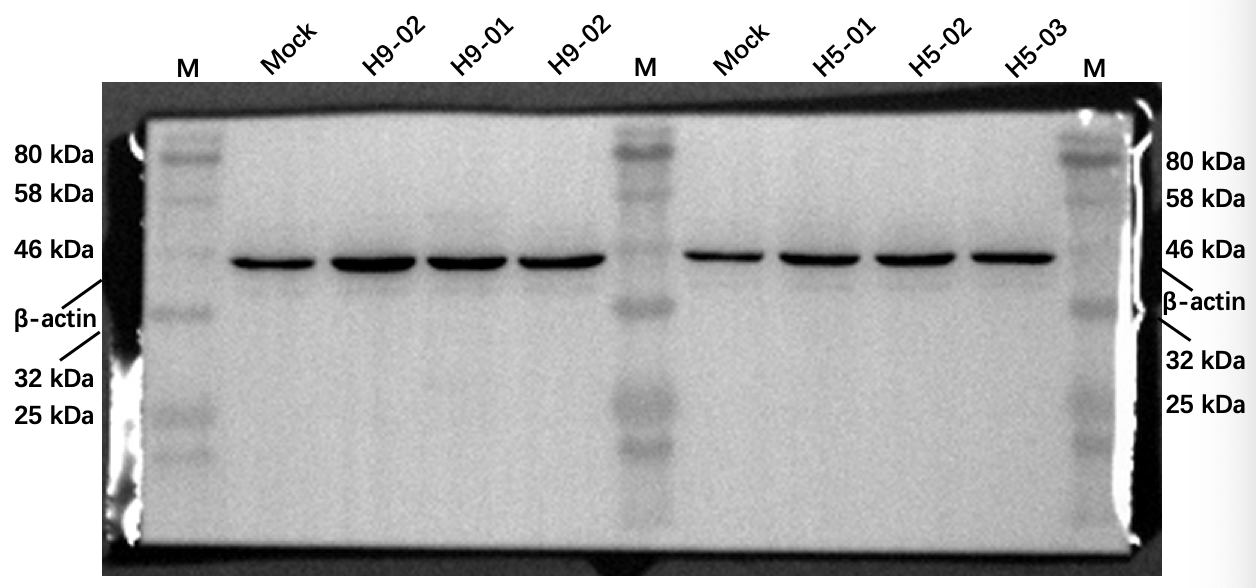


**Fig 4 Original blot of figure 4 in text, Western-blotting analysis of β-actin protein from H5 and H9 subtypes IAVs infected samples:** Lysates of chicken embryo fibroblasts (CEF) infected with IAVs at MOI of 0.01 for 12 h–15 h were incubated with a monoclonal antibody (mAb) against β-actin. Bands were visualized using a chemiluminescence imaging analysis system after incubation with horse radish peroxidase (HRP)-labeled secondary antibodies. H9-01: A/Chicken/Shanghai/F/98; H9-02: A/Chicken/Taixing/10/2010; H5-01: A/Mallard/Huadong/S/2005; H5-02: A/Duck/Huadong/wx1205/2016; H5-03: A/Goose/Huadong/yz1111/2016.


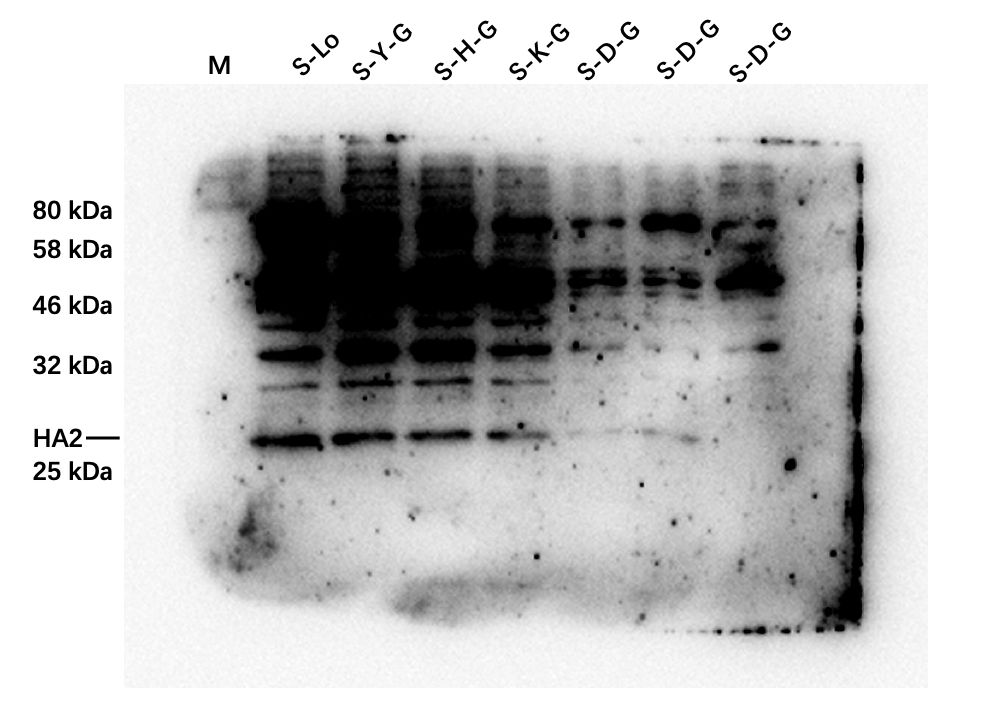


**Fig 5 Original blot of figure 7 in text, Western-blotting analysis of HA2 protein from mutant viruses infected samples:** Lysates of chicken embryo fibroblasts (CEF) infected with mutant viruses at MOI of 0.01 for 12 h–15 h were incubated with a primary antibody against the 14^th^ peptide. Bands were visualized by a chemiluminescence imaging analysis system after incubation with horse radish peroxidase (HRP)-labeled secondary antibodies.


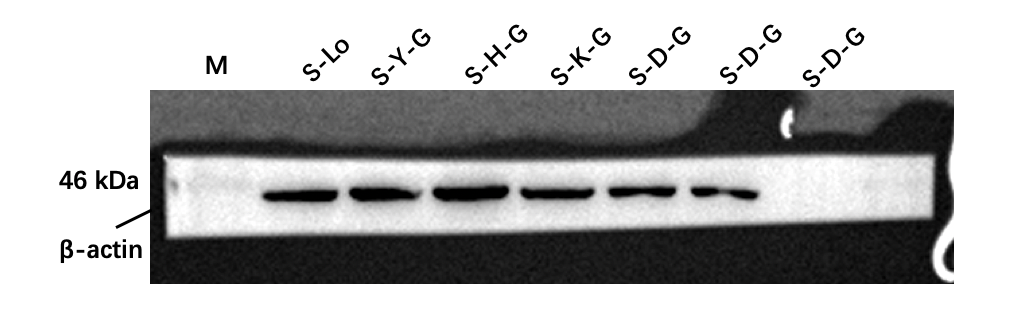


**Fig 6 Original blot of figure 7 in text, Western-blotting analysis of β-actin protein from mutant viruses infected samples:** Lysates of chicken embryo fibroblasts (CEF) infected with mutant viruses at MOI of 0.01 for 12 h–15 h were incubated with a primary antibody against the 14^th^ peptide and monoclonal antibody (mAb) against β-actin. Bands were visualized by a chemiluminescence imaging analysis system after incubation with horse radish peroxidase (HRP)-labeled secondary antibodies.
